# The Luminal Progenitor Compartment of the Normal Human Mammary Gland Constitutes a Unique Site of Telomere Dysfunction

**DOI:** 10.1016/j.stemcr.2013.04.003

**Published:** 2013-06-04

**Authors:** Nagarajan Kannan, Nazmul Huda, LiRen Tu, Radina Droumeva, Geraldine Aubert, Elizabeth Chavez, Ryan R. Brinkman, Peter Lansdorp, Joanne Emerman, Satoshi Abe, Connie Eaves, David Gilley

**Affiliations:** 1 Terry Fox Laboratory, British Columbia Cancer Agency, Vancouver, BC V5Z 1L3, Canada; 2 Department of Medical and Molecular Genetics, Indiana University School of Medicine, Indianapolis, IN 46202-5251, USA; 3 European Research Institute for the Biology of Ageing, University Medical Center Groningen, and University of Groningen, Antonius Deusinglaan 1, 9713 AV Groningen, The Netherlands; 4 Department of Cellular and Physiological Sciences, University of British Columbia, Vancouver, BC V6T 1Z3, Canada

## Abstract

Telomeres are essential for genomic integrity, but little is known about their regulation in the normal human mammary gland. We now demonstrate that a phenotypically defined cell population enriched in luminal progenitors (LPs) is characterized by unusually short telomeres independently of donor age. Furthermore, we find that multiple DNA damage response proteins colocalize with telomeres in >95% of LPs but in <5% of basal cells. Paradoxically, 25% of LPs are still capable of exhibiting robust clonogenic activity in vitro. This may be partially explained by the elevated telomerase activity that was also seen only in LPs. Interestingly, this potential telomere salvage mechanism declines with age. Our findings thus reveal marked differences in the telomere biology of different subsets of primitive normal human mammary cells. The chronically dysfunctional telomeres unique to LPs have potentially important implications for normal mammary tissue homeostasis as well as the development of certain breast cancers.

## Highlights

• Normal human mammary gland luminal progenitors (LPs) have very short telomeres
• LP nuclei selectively exhibit telomere-associated DNA damage responses
• LPs have selectively elevated hTERT expression and telomerase activity
• These LP features may play a role in mammary tissue homeostasis and transformation

## Graphical Abstract

**Figure:**
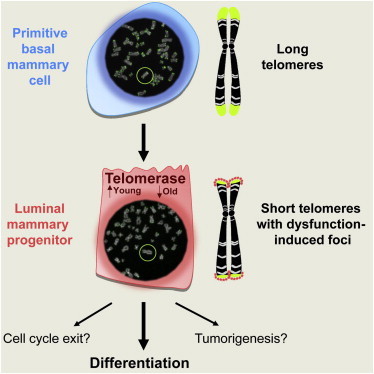

## Introduction

Chromosome ends, referred to as telomeres, contain repeat sequences (TTAGGG)_n_ and associated proteins that protect cells from the formation of chromosome end-to-end fusions (Artandi and DePinho, 2010). In normal tissues, such as in the hematopoietic system, where cell turnover is high and continuous throughout life, telomeres are maintained in the most primitive cells at relatively long lengths and then become progressively shorter as the cells differentiate through multiple amplifying divisions and with age (Aubert and Lansdorp, 2008). Epithelial tissues, including the mammary gland in both humans and mice, also undergo extensive turnover, and recent studies indicated that this may involve a similarly organized hierarchical differentiation process (Eirew et al., 2008; Visvader, 2009). To date, analysis of telomere length regulation in normal mammary epithelial cells has been limited to reports of shorter telomeres in luminal cells (Kurabayashi et al., 2008; Meeker et al., 2004), and human telomerase reverse transcriptase (hTERT) messenger RNA (mRNA; Kolquist et al., 1998) in histological sections. However, the presence of telomere fusions was noted in primary mammary epithelial cells after their extensive passage in vitro (Romanov et al., 2001), and we recently reported that telomere-dysfunction-specific chromosomal fusions are common in early-stage breast cancers (Tanaka et al., 2012). Here we show that phenotypically separable compartments of normal human mammary epithelial cells with distinct biological properties have markedly different telomere lengths and telomerase activities. Interestingly, a phenotype that is highly enriched in luminal progenitors (LPs) is uniquely characterized by critically short telomeres, frequent evidence of a telomere-specific DNA damage response (DDR), and an age-related decrease in telomerase activity.

## Results and Discussion

### Normal Human Mammary LPs Possess Short Telomeres

**Figure 1:**
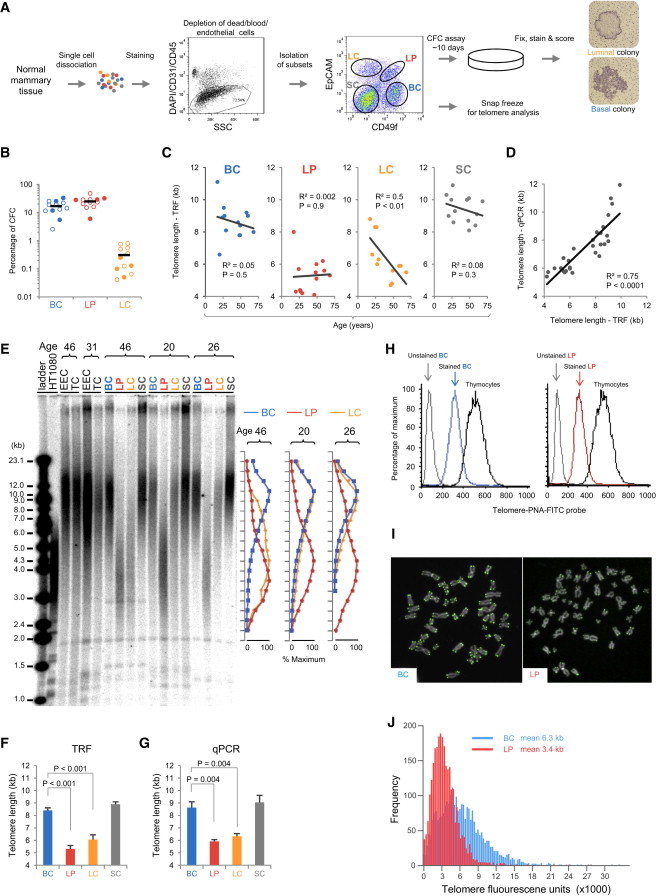
Telomere Length Alterations in Different Subsets of Cells Present in Normal Human Breast Tissue (A) Process for isolating and characterizing the four subsets of cells studied (after removal of CD45^+^ hematopoietic and CD31^+^ endothelial cells), showing examples of colonies obtained from cells in the LP (above) and BC (below) fractions. BCs are defined by their CD49f^hi^EpCAM^neg/low^ phenotype and contain bipotent and self-renewing mammary stem cells identified by in vivo transplantation assays (Eirew et al., 2008; Lim et al., 2009). BCs also include CFCs that generate mixtures of luminal and myoepithelial cells (bipotent CFCs) as well as CFCs that generate pure myoepithelial cell colonies (myoepithelial CFCs) and “mature” myoepithelial cells that do not have CFC activity. The CD49f^hi^EpCAM^hi^ fraction contains mammary cells with features of luminal cells as well as CFCs that produce pure luminal cell colonies at high frequency (Raouf et al., 2008) and accordingly is referred to here as the LP fraction. The third subset of cells have an CD49f^lo^EpCAM^hi^ phenotype and are referred to as LCs because they are devoid of growth activity in either in vivo or in vitro assays and are considered to be developmentally downstream of LPs. Note the color code adopted for all subsequent figures: blue, BCs; red, LPs; orange, LCs; gray, SCs. (B) CFC frequencies in the phenotypically defined BC, LP, and LC fractions of 12 of the samples, including several used for telomere length measurements by southern blot (closed circles). (C) TRF length measurements for the different subsets as a function of donor age (n = 13). (D) Correlation of the average TRF length measured by southern blot and qPCR analysis of 28 different DNA extracts (12 different donor samples). (E) Representative southern blot showing the spread of TRFs obtained from BCs, LPs, LCs, and SCs, as well as from initial unseparated mammary cells (epithelial-enriched cells [EECs] and total breast cells [TCs]), and HT1080, a human tumorigenic fibrosarcoma cell line with very short telomeres. To the right are distributions of TRFs shown as a percentage of the maximum telomere probe signal for the examples represented in the adjacent southern blot. (F) TRF lengths for each of the four subsets from southern blots (mean ± SEM, 13 different donor samples). (G) qPCR telomere lengths measured for each of the four subsets (mean ± SEM, seven different donor samples); p values were calculated using a paired two-tailed Student’s t test. (H) Representative Flow-FISH plots of telomere-specific fluorescein-conjugated (CCCTAA)_3_ PNA fluorescence in individually assessed BCs and LPs compared with a spiked-in calf thymocyte standard. The average modal values from three matched pairs of BCs and LPs were 8.9 ± 0.4 and 7.9 ± 0.3, respectively (p < 0.05, one-tailed Student’s t test). (I) Q-FISH-stained chromosomes of representative BC and LP metaphases. The DAPI channel is labeled as gray and the telomere probe channel is labeled as green. (J) Frequency distribution of telomere fluorescence levels measured on 15 metaphases from 3-day cultures of BCs and LPs (treated with colcemid for 2 hr). Mean telomere length was calculated as described previously (Poon et al., 1999).

**Figure 2:**
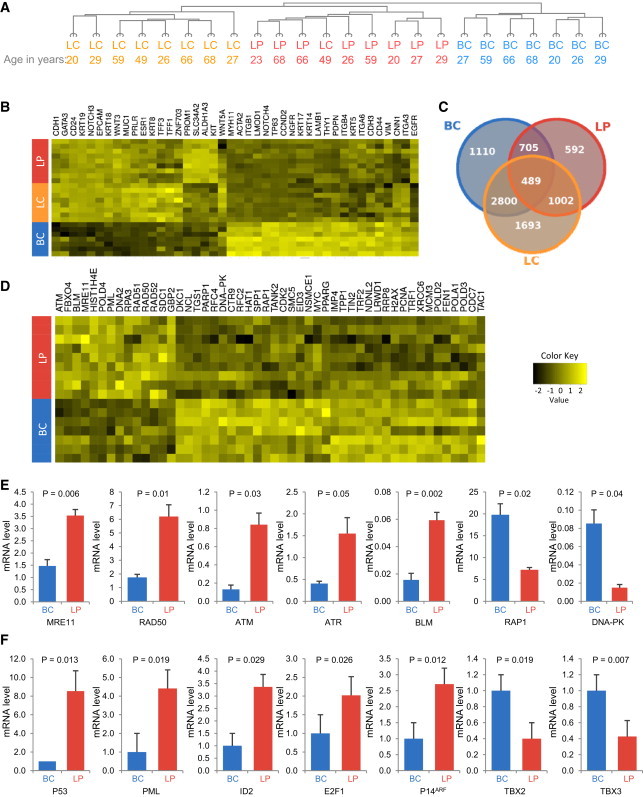
Distinct Expression Profiles of Telomere-Associated Genes in Normal Mammary Epithelial Subpopulations (A) Unsupervised hierarchical clustering of gene-expression data from microarray analyses of purified cell fractions. (B) Unsupervised hierarchical clustering of gene-expression profiles of known lineage markers. A heatmap (yellow = upregulated, black = downregulated) demonstrates the relative expression of each marker gene. (C) Venn diagram showing the numbers of significantly (p < 0.05) differently expressed genes between different subset pairs. (D) Telomere-associated gene-expression profiles of the BC and LP subsets. The top 50 differentially expressed telomere-associated genes identified by a search in GO were used to classify BCs and LPs using an unsupervised clustering algorithm. (E) qRT-PCR of DDR gene transcript levels from four samples. (F) qRT-PCR of senescence-associated gene transcripts from five to eight samples. Error bars represent mean ± SEM; p values were determined by a paired two-tailed Student’s t test.

**Figure 3:**
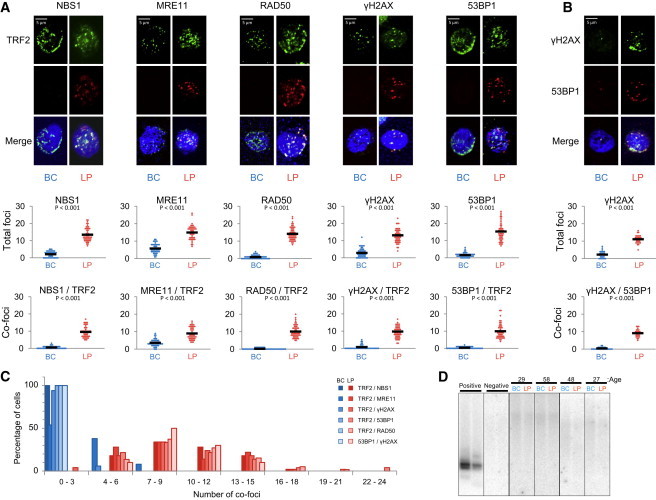
Detection of TIFs in the Nuclei of Normal Human Mammary LPs but Not BCs (A) Upper panels: Representative images of single BC and LP nuclei, showing differential colocalization of TRF2 with NBS1, MRE11, RAD50, γ-H2AX, and 53BP1. Middle panels: The total number of TIFs per nucleus (>50 nuclei examined for each DDR gene). Lower panels: The frequency of affected BC and LP nuclei, with average values indicated by the black bars in each case. (B) Representative images of BCs and LPs, showing differential colocalization of γ-H2AX with 53BP1. The total numbers of γ-H2AX/53BP1 foci in LPs and BCs are presented in dot plots and the average values are shown as black bars. (C) Frequency of BCs and LPs displaying colocalized foci of NBS1, MRE11, RAD50, γ-H2AX, and 53BP1 with TRF2. (D) TAR-Fusion PCR performed on BCs and LPs isolated from four different samples obtained from donors of various ages. Positive control: DNA from human BJ E6/E7 foreskin fibroblast cells with telomere crisis. Negative control: DNA from primary human BJ foreskin fibroblast.

**Figure 4:**
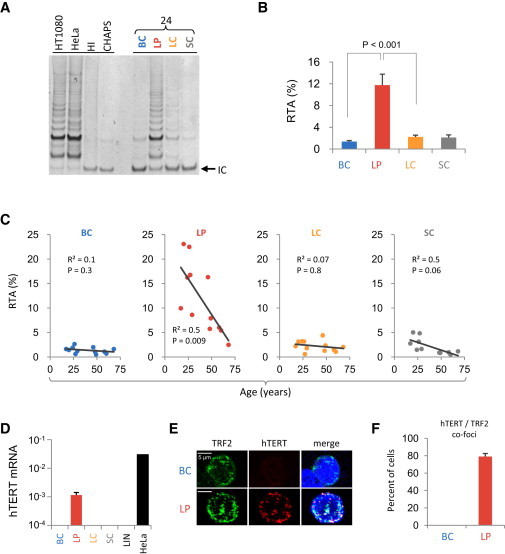
Telomerase Expression and Activity in Normal Human Mammary Epithelial Cell Subsets (A) Representative gel comparing the telomerase activity of BCs, LPs, LCs, and SCs assessed by the TRAP assay using nondenatured lysates of HT1080 and HeLa cells as a positive control, and heat-inactivated (HI) cellular lysate and CHAPS detergent buffer in the absence of cellular lysate as negative controls. IC, internal control. (B) Comparison of TRAP activity measurements obtained on all four subsets from all 12 samples analyzed expressed as a percentage of the activity in HeLa cells. Values shown are the mean ± SEM. (C) Distribution of the individual telomerase activity measurements shown in (B), but displayed for each subset as a function of the age of the donor. The p value shows age association significance. (D) *hTERT* transcript levels measured by qRT-PCR analyses of each of the four subsets by comparison with HeLa cells. Extracts of BCs, LCs, SCs, and mammary DAPI^−^CD31^+^CD45^+^ (LIN^−^) cells (three samples) did not contain detectable RNA levels. Values shown for LPs are the mean ± SEM from the same three samples. (E) Representative confocal laser microscope images of the nuclei of purified permeabilized BCs and LPs stained with SYTOX Blue and differently labeled antibodies to hTERT (in red) or TRF2 (green), or both. Colocalized foci are yellow. (F) Frequency of BC and LP nuclei that demonstrated colocalized hTERT^+^TRF2^+^ foci (yellow). Values are the mean ± SEM of multiple nuclei from three different donors (p < 0.0001, paired two-tailed Student’s t test).

To examine telomere length in different compartments of normal human breast tissue and possible age-related changes, we isolated four phenotypically distinct subsets of cells at high purities (>95%) from different normal reduction mammoplasty tissue samples obtained from donors of different ages and analyzed them directly, without culture (Figure 1A; Table S1 available online). We examined four subsets of cells: (1) a basal epithelial cell (BC) subset that is highly enriched in cells with bipotent as well as myoepithelial clonogenic activity in vitro (Figure 1B), (2) an LP subset that is similarly enriched in cells with luminal clonogenic activity in vitro (Figure 1B), (3) a third mammary epithelial cell subset that contains exclusively mature luminal cells (LCs) with no clonogenic activity (Figure 1B), and (4) a population consisting of nonepithelial stromal cells (SCs) that are still prominent after the hematopoietic and endothelial cells are removed (Figure 1A). Extracts of each of these four highly purified cell populations were analyzed for telomere length both by quantitative phosphorimage scanning of telomere restriction fragment (TRF) lengths in southern blots (Aviv et al., 2011; Cawthon, 2009) and by quantitative PCR (qPCR; Aviv et al., 2011; Cawthon, 2009; Figures 1C–1G). To evaluate telomere length values for individually analyzed cells in the BC and LP subsets, we used flow-fluorescent in situ hybridization (Flow-FISH) to examine freshly isolated cells (Rufer et al., 1998; Figure 1H) and quantitative FISH (Q-FISH) to examine metaphases obtained on the proliferating progeny of LPs and BCs present in 3-day adherent cultures of these cells (Poon et al., 1999; Figures 1I and 1J). The striking finding from all of these analyses is the very short average telomere length that uniquely characterizes LP cells, regardless of the age of the donor. The southern data imply that some chromosomes within the LP population likely contain TRFs <3 kb in length (Figure 1F) and this was also evident from the Q-FISH data (Figure 1J). This telomere length has been associated with telomere dysfunction and cell death in the absence of telomerase (Begus-Nahrmann et al., 2012; Hemann et al., 2001). By comparison, the average telomere length of the BCs is considerably longer (∼6–8 kb) and also age independent. However, the importance of age as a variable is evident in the terminally differentiated LCs (Figure 1C). Analyses of the matching SCs showed that these cells contain even longer telomeres than any of the mammary epithelial cells (on average ∼9 kb by southern blot analysis), and also do not show significant changes in telomere length with age (Figures 1C and 1E–1G).

The age-related changes in telomere length observed in the isolated LCs were masked in southern analyses of DNA extracts obtained from unseparated breast tissue (Figure 1E, lane marked TC). This likely reflects the low representation of the LCs in whole breast tissue relative to the predominance of other cells (Figure 1A) regardless of donor age. The discovery of short telomeres specific to the LP compartment is notable given that a high proportion of cells with an LP phenotype (up to 48%; Figure 1B) can execute multiple divisions in vitro.

### LPs Display Evidence of a Telomere-Associated DDR without Telomere Fusions

To determine whether the short telomeres characteristic of normal human LPs are sufficient to elicit a DDR, we first performed microarray analyses on RNA extracted from BC, LP, and LC populations purified from young (premenopausal, 20–49 years old, n = 6) and older donors (postmenopausal, 59–68 years old, n = 3). Unsupervised hierarchical clustering of the data demonstrated that the cell subset, but not donor age, was a strong classifier (Figure 2A). Examination of genes associated with mammary cell differentiation confirmed both expected and novel associations (Figures 2B and 2C). For example, BCs expressed the highest levels of transcripts for smooth muscle actin (*ACTA2*), *TP63*, *NOTCH4*, keratin 4 (*KRT4*), *KRT5*, *KRT14*, *KRT17*, vimentin, *THY1*, and *CD44*, which are all established BC markers. Similarly, LPs and LCs expressed higher levels of transcripts than the BCs for many known luminal markers (*CD24*, *MUC1*, *KRT18*, *KRT19*, *EPCAM*, *NOTCH3*, and *GATA3*). LPs also expressed the highest levels of prominin1 (*CD133*), *KIT*, and *ALDH1A* transcripts, as predicted by the literature (Eirew et al., 2012; Lim et al., 2009; Raouf et al., 2008). LCs expressed the highest levels of *ZNF703* (Holland et al., 2011), estrogen receptor-α (*ESR1*), trefoil factor 3 (*TFF3*), and *KRT8*, which have been associated with luminal-type mammary cancers (Lim et al., 2009). The progenitor-enriched BC and LP subsets also displayed increased epidermal growth factor receptor (*EGFR*) transcripts consistent with their requirement for EGF to support their clonogenic activity in vitro.

Using the Gene Ontology (GO) database (http://www.geneontology.org), we identified 50 telomere-associated genes that are expressed in LPs at significantly different levels than in BCs (Figure 2D). Expression of 13 of these genes was elevated in LPs, and these included several DDR genes (*MRE11*, *RAD50*, *ATM*, *ATR*, and *BLM*). ATM is the primary activator of DNA double-strand break repair and requires the early initiation factor MRN complex that is composed of MRE11, RAD50, and NBS1 (Uziel et al., 2003). Higher expression of *MRE11*, *RAD50*, *ATM*, *ATR*, and *BLM* in LPs as compared with BCs was confirmed in quantitative RT-PCR (qRT-PCR) analyses of additional samples (Figure 2E). In contrast, *RAP1* and *DNA-PK* were more highly expressed in the BCs.

DDR gene products in the MRN complex have identified roles in the repair of DNA double-strand breaks (van den Bosch et al., 2003) and have also been reported to localize at telomeres following induction of telomere dysfunction in various cell line models (Stracker and Petrini, 2011). To determine whether LPs and BCs would show differences in telomere-associated DDR sensors, we fixed freshly isolated LPs and BCs; stained them with antibodies to NBS1, RAD50, γ-H2AX, 53BP1, MRE11, and TRF2 (a protein that specifically localizes to telomeres [Tomlinson et al., 2006] and also served as a permeabilization control); and then examined individual nuclei by confocal imaging. Importantly, in spite of the lower levels of *TRF2* transcripts in LPs, TRF2 protein could be readily detected immunochemically at telomere foci in these cells as well as in BCs. We found that NBS1, RAD50, γ-H2AX, and 53BP1 were essentially undetectable in the nuclei of BCs but strongly present in the nuclei of all LPs examined, where they were consistently colocalized with TRF2 (∼10 telomere dysfunction-induced foci [TIFs] per LP nucleus; Figures 3A–3C). MRE11 was detectable and colocalized with TRF2 in some BC nuclei, but significantly less so than in LP nuclei (p < 0.00001; Figure 3). The MRE11 present at the telomeres of BCs may reflect the known role of MRE11 in conventional telomere maintenance (Zhu et al., 2000). In contrast, the high levels of γ-H2AX, 53BP1, and other early DDR proteins at the telomeres of LPs suggests that throughout adult life, at least some of these cells experience biologically significant consequences of telomere dysfunction.

Interestingly, however, neither telomere-associated repeat (TAR)-fusion PCR analyses (Tanaka et al., 2012) of freshly isolated LPs and BCs (Figure 3D) nor examination of the metaphases prepared for the Q-FISH analyses from the LPs (and BCs) revealed any evidence of telomere fusions (Figure 1I). This suggests that the very short telomeres of at least some cells in the LP compartment must prevent their further progression through the cell cycle, and possibly explains why higher frequencies of LPs with in vitro clonogenic activity have not been achievable (Eirew et al., 2008; Raouf et al., 2008; Figure 1B). It was previously suggested that maintenance of mammary epithelial cells in vitro leads to a progressive increase in TIFs, with the induction of senescence following the accumulation of five or more TIFs per cell (Kaul et al., 2012). As with replicative cellular senescence, telomere shortening and cellular responses to consequent DNA damage may thus act primarily as a tumor-suppressive mechanism in mammary LPs (Campisi, 2011).

Although senescence programs relevant to human cells in vivo have not yet been established, increased lysosomal content and altered expression of several genes have both been associated with senescence in various cell line models. Fluorescence-activated cell sorting (FACS) analysis of LPs and BCs with LysoTracker dye showed the LPs to have a higher lysosomal content (Figure S1). Likewise, higher expression of *P14*, *P53*, *PML*, *ID2*, and *E2F1*, and decreased expression of *TBX2* and *TBX3* (genes designated as senescence associated in GO) were also evident in our present and previously published transcriptome data sets (Figures S2 and S3). These gene-expression findings were confirmed by qRT-PCR (Figure 3F).

Thus, LPs may be predisposed to events that confer oncogenic properties on cells that acquire critically short telomeres, but appear to be protected from such events, at least to some extent, by activation of mechanisms that cause them to exit the cell cycle and/or senesce. The short telomere length of the LPs and their high frequency of TIFs may be particularly relevant in light of recent studies that implicated these cells in the development of BRCA1-associated breast cancers, and showed that malignant cells had a transcriptional profile similar to that of normal LPs even though they exhibited a BC phenotype (Lim et al., 2009; Proia et al., 2011). Since an association of short telomeres with increased sensitivity to other sources of DNA damage has been reported (Berardinelli et al., 2012; Wong et al., 2000), it will be of interest to determine whether this extends to LPs or whether other mechanisms override such a relationship.

### hTERT Expression and Telomerase Activity Are Upregulated in LPs but Decline with Age

hTERT, the catalytic (and limiting) component of telomerase activity, is absent from most differentiated somatic human cells (Shay and Bacchetti, 1997), including normal breast tissue (Shay and Bacchetti, 1997; Tanaka et al., 2012). Given our finding of short telomeres and evidence of a DDR in the LPs, we asked whether hTERT expression and activity are detectable in these cells (Figure 4). Using the telomere repeat amplification protocol (TRAP) assay (Herbert et al., 2006), we found that the level of telomerase activity in LPs approached that previously observed in telomerase-dependent malignant HeLa cells (Shay and Wright, 2010), but detected no activity in the three other subsets (Figures 4A and 4B). Interestingly, the telomerase activity in LPs was particularly elevated in younger women and then declined ∼5-fold with age (Figure 4C), consistent with the parallel age-associated reduction of LC telomere length (Figure 1C). qRT-PCR measurements of *hTERT* mRNA levels (Figure 4D) and confocal imaging of telomere-associated hTERT expression (Figures 4E and 4F) confirmed that hTERT expression in the LPs was consistently (∼80% of LPs) and selectively elevated (Figure 4D). These studies also show that the hTERT present in LPs is associated with their telomeres, as evidenced by the frequency of intranuclear hTERT^+^TRF2^+^ foci seen (approximately three foci per nucleus in ∼80% of the LPs; Figures 4E and 4F).

The decreasing levels of telomerase activity seen in the LPs of older women is not inconsistent with the age-independent telomere lengths of LPs because the latter are likely sustained long term by their continuous derivation from more primitive cell types in the BC compartment (Eirew et al., 2008). Additionally, LP cells with critically short telomere lengths may be eliminated by exit from the cell cycle through a senescence pathway (Campisi, 2011; Hemann et al., 2001). However, the decreasing levels of telomerase activity seen in the LPs of older women may affect their ability to generate LCs, since LPs are thought to be LC precursors. On the other hand, the relatively high levels of telomerase activity found in LPs may have novel, telomere-length-independent roles, as suggested by others (Elenbaas et al., 2001; Hemann et al., 2001; Kiyono et al., 1998; Park et al., 2009; Sharma et al., 2003; Smith et al., 2003).

Our findings also raise the interesting possibility that the changes in telomere length regulation and their sequelae in the LP subset of cells in the normal human mammary gland may occur in the transit-amplifying compartment of other epithelial cell populations where an analogous differentiation hierarchy exists. It will thus be of interest to elucidate the intrinsic and exogenous factors that appear to link these changes to the control of differentiation in normal human mammary cells.

## Experimental Procedures

### Cells and Colony-Forming Cell Assays

Histologically confirmed normal, anonymized tissue from 37 women undergoing cosmetic reduction mammoplasties (Table S1) was obtained according to procedures approved by the University of British Columbia Ethics Board and then processed and cryopreserved in 6% DMSO-containing medium (Stingl et al., 2005). Thawed cells were labeled with different fluorochrome-conjugated antibodies (listed in Table S2) and DAPI to enable highly purified subsets to be isolated by FACS (>95% after a single sort and >98% after a second re-sort, as determined by further flow cytometric analysis) using either an Influx-II or ARIA FACS (Becton Dickinson) as described in detail previously (Eirew et al., 2008). The same results were obtained from both single- and double-sorted cells and therefore were pooled. Colony-forming cell (CFC) assays were performed in dishes precoated with 1.6% Matrigel (BD Biosciences) in SF7 medium with 5% fetal bovine serum (STEMCELL Technologies), 20 μM ROCK inhibitor (Reagents Direct), and irradiated feeders.

### Telomere Length Measurements

Genomic DNA isolated using QIAamp DNA Blood Mini Kits (QIAGEN) was digested with *Hae*III, *Hinf*I, and *Rsa*I, and DNA fragments were separated by electrophoresis on 0.8% SeaKem LE agarose gels (Lonza) and hybridized with ^32^P-labeled (TTAGGG)_4_. Blots were scanned with a PhosphorImager (Molecular Dynamics) and mean TRF lengths were determined (Huda et al., 2007, 2009). qPCR measurements (Cawthon, 2009) were performed in triplicate in 15 μl reaction volumes with reaction conditions of 10 min at 94°C, two cycles for 10 s each at 94°C and 15 s at 49°C, followed by 35 cycles at 94°C for 10 s each, 62°C for 15 s, and 74°C for 30 s with fluorescence signal acquisition. Automated multicolor Flow-FISH was performed as previously described (Baerlocher et al., 2006). Q-FISH with Cy3-labeled (CCCTAA)_3_ peptide nucleic acid (PNA) probes and analysis of telomere length from digital images were performed as previously described (Poon et al., 1999) on metaphases obtained from 3-day cultures (CFC assay conditions) initiated with matched LPs and BCs (15 metaphases each) isolated from one of the samples used for southern analysis.

### TAR-Fusion PCR

Genomic DNA was isolated by the salt precipitation method, and TAR-Fusion qPCR was carried out as previously described (Tanaka et al., 2012) using a two-step touchdown PCR in a 20 μl reaction mixture including 50 ng of DNA, multiple primers, 10% 7-Deaza-dGTP (Roche Diagnostics), and Advantage GC Genomic LA Polymerase Mix (Clontech). To calculate the percentage of telomere fusions detectable by TAR-Fusion PCR, we used the general equation *C*(*n*, 2) *+ n*, where *n* is the number of unique chromosomal ends. The total number of detectable fusion combinations was 154 and the coverage rate was 14.2%.

### TRAP Assays

TRAP assays were performed on 3-[(3-cholamidopropyl) dimethylammonio]-1-propanesulfonate (CHAPS) extracts containing 0.25 μg of total protein using the TRAPeze telomerase detection kit (Millipore), and PCR products were resolved by electrophoresis in 12.5% polyacrylamide gels and visualized by SYBR Green phosphorimaging as previously described (Herbert et al., 2006). TRAP activity was then quantified using a LightCycler 480 II (Roche) and cross-point (Cp) values were determined using the second derivative maximum method. These values were then converted to protein concentrations (μg/μL) as an indicator of telomerase activity by the fit-points method using LightCycler 480 software (1.5.0; Roche) and an HT1080 standard curve, and then expressed as relative values using an extract of HeLa cells as a reference.

### Confocal Laser Microscopy and Image Analysis

Cells were cytospun onto slides, rinsed with PBS, fixed with 4% formaldehyde (Thermo Scientific, Rockland, IL, USA), permeabilized with 0.2% Triton X-100 (Sigma-Aldrich, St. Louis, MO, USA), and blocked with 6% BSA (Fraction V; Fisher Scientific) in PBS. The cells were then incubated with various antibodies at room temperature for 1 hr, washed in PBS, and incubated with secondary antibodies (listed in Table S2) as recommended by the manufacturer. After further washing with PBS, nuclei were labeled with SYTOX Blue (Invitrogen), mounted with Qmount (Invitrogen), and imaged on an Olympus FV1000MPE confocal/multiphoton microscope using FV-ASW 3.0 Viewer software.

### qRT-PCR

Complementary DNA (cDNA) was generated from Trizol extracts using the Quantiscript RT Fastlane cDNA kit (QIAGEN). The primers used for qRT-PCR are listed in Table S3. Samples were subjected in triplicate to 40 amplification cycles (10 s at 95°C, 20 s at 60°C, and 30 s at 72°C). Two negative controls (one in which no cDNA template was added and one in which no RT treatment was applied) were included in each experiment. Gene transcript levels were calculated using the ΔΔCt method (Livak and Schmittgen, 2001), with TATA box-binding protein (*TBP*) expression used as a loading control.

### Microarray Analysis

RNA in Trizol was repurified using the RNeasy Micro kit (QIAGEN), and preparations with an RNA integrity number of ≥8.0 were prepared using the Agilent One-Color Microarray-Based Exon Analysis Low Input Quick Amp WT Labeling Kit v1.0. Aliquots of 25 ng total RNA were used to generate Cyanine-3-labeled complementary RNA, which was then hybridized on 8 × 60K Agilent Whole Human Genome Oligo Microarrays (Design ID 028004) and scanned at 3 μm resolution in an Agilent DNA microarray scanner and data processed using Agilent Feature Extraction 10.10. The processed signal was quantile normalized with Agilent GeneSpring 11.5.1 and analyzed using the open-source language R.

## Accession Numbers

The GEO accession number for the transcriptome data sets reported in this paper is GSE37223.
