# Supplementary material for: The Luminal Progenitor Compartment of the Normal Human Mammary Gland Constitutes a Unique Site of Telomere Dysfunction
Source: Stem Cell Reports. 2013 Jun 4;1(1):28–37. doi: 10.1016/j.stemcr.2013.04.003 (PMC3757746; doi:10.1016/j.stemcr.2013.04.003)
Supplement: Document S1. Tables S1–S3 and Figures S1–S3 [file mmc1.pdf]

Stem Cell Reports, Volume *1*

Supplemental Information

The Luminal Progenitor Compartment of the Normal Human Mammary Gland Constitutes a Unique Site of Telomere Dysfunction

Nagarajan Kannan, Nazmul Huda, LiRen Tu, Radina Droumeva, Geraldine Aubert, Elizabeth Chavez, Ryan R. Brinkman, Peter Lansdorp, Joanne Emerman, Satoshi Abe, Connie Eaves, and David Gilley

Inventory of Supplemental Information

- Figure S1. Lysosome Content Analysis by FACS, Related to Results and Discussion
- Figure S2. Differential Expression of Telomere-associated Genes in Lim et al, Related to Results and Discussion
- Figure S3. Differential Senescence-associated Genes in the Datasets Described in this Paper and in Lim et al, Related to Results and Discussion
- Table S1. Summary of the Reduction Mammoplasty Samples Used in this Study, Related to Results and Discussion
- Table S2. Antibodies and their Application, Related to Experimental Procedures
- Table S3. Primers Used for qRT-PCR Analysis, Related to Experimental Procedures

Figure S1. Lysosome content analysis by FACS. Dissociated mammary single cells were incubated with 50nM live cell-permeable LysoTracker (Invitrogen) dye for 30 min at 37°C in dark. Cells were then immediately stained for cell surface markers and analyzed by FACS. Panel (A) shows fold increase in mean LysoTracker fluorescence intensity (MFI) in LPs compared to BCs isolated from 6 different samples. P values determined using the paired 2-tailed Students t-test. Error bar shows MFI ± SEM. Panel (B) shows representative FACS plot.

A

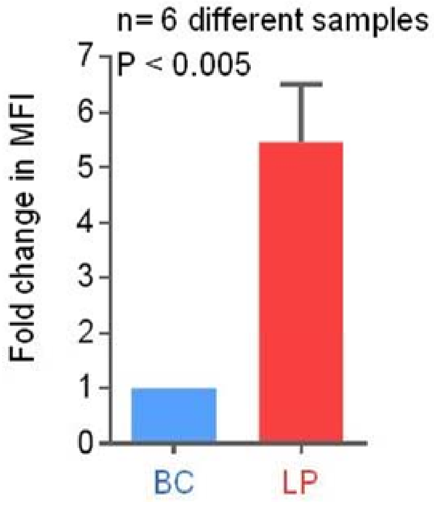

B

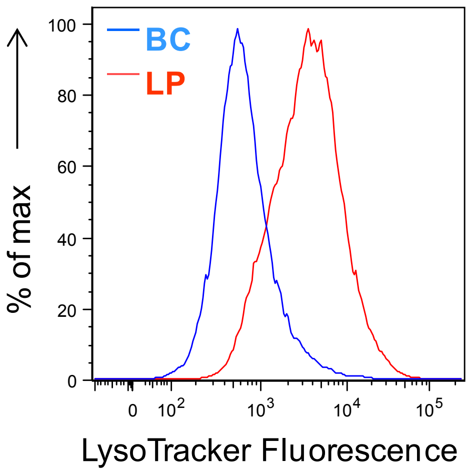

Figure S2. Differential expression of telomere associated genes in Lim et al. Commonly found genes in the dataset described in this paper and Lim et al dataset are indicated by arrow.

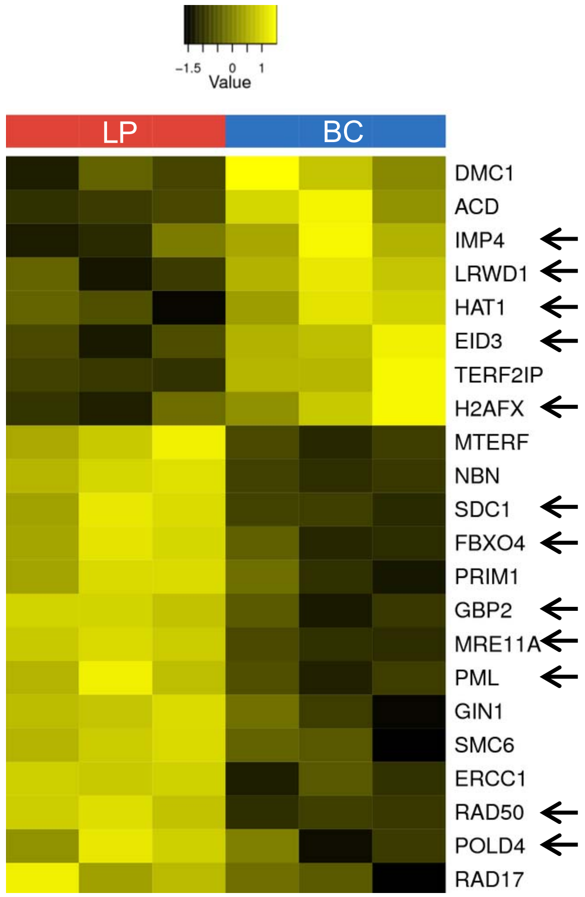

Figure S3. Differential expression of senescence-associated genes in dataset described in this study and Lim et al dataset. Commonly differentially regulated genes are indicated by arrow.

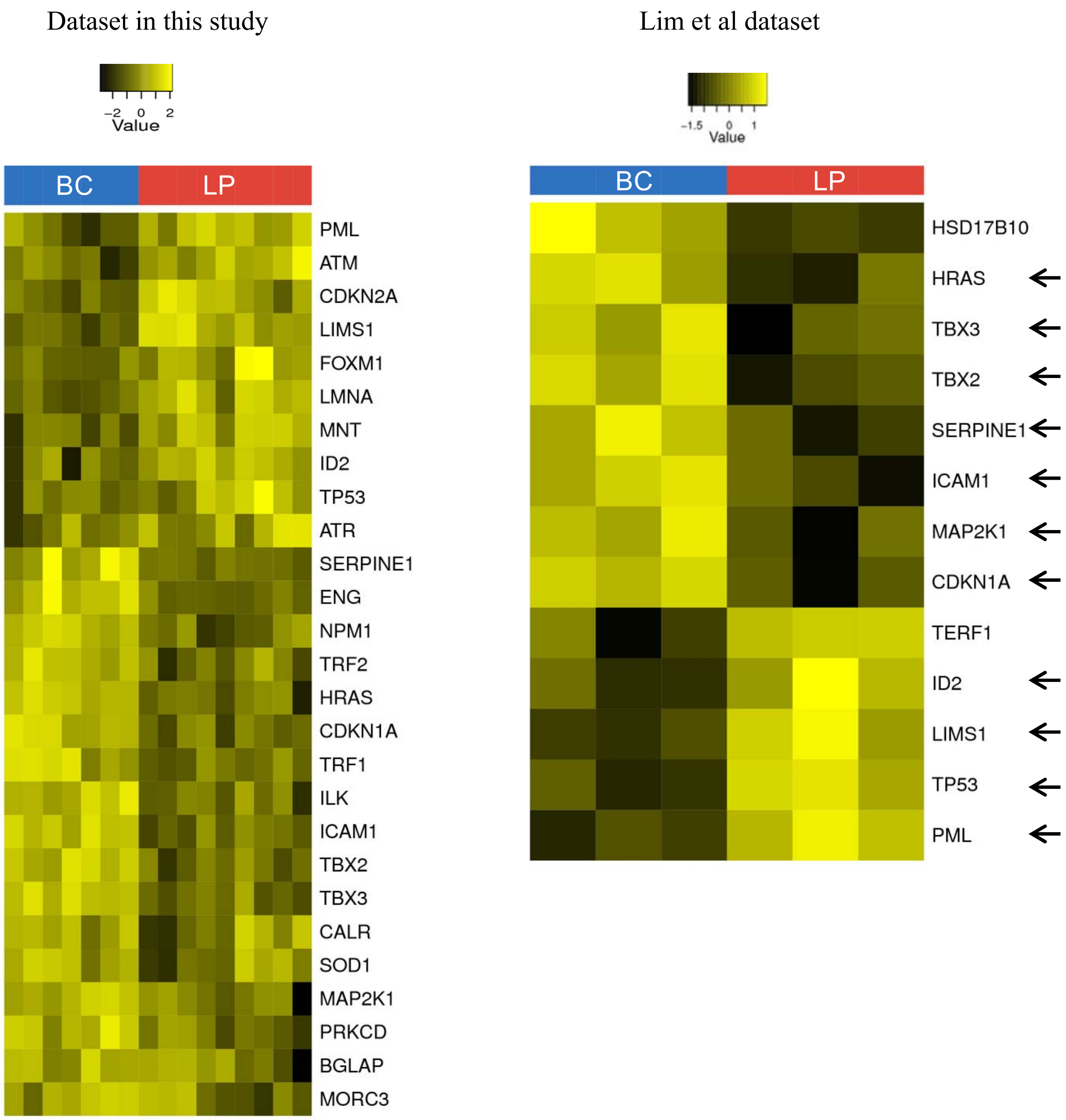

Table S1. Summary of reduction mammoplasty samples used in this study

Patient ID	Age (Years)	Meno-pausal status		Hormonal status		Southern blot (TRF)	Telo qPCR	Flow-FISH	Meta-phase Q-FISH	TAR-Fusion PCR	CFC	FACS purificati on type	Micro array	QPCR validat ion	Colocalization studies
		Pre	Post	Secre-tory	Prolif-erative										
23-09	17	+		+		+	+				+	Single			
189-03	18	+		+		+					+	Single			
83-09	20	+		+								Single	+		
25-10	24	+		+		+	+				+	Single			
21-10	26	+		+		+			+			Single	+		
60-10	27	+			+	+	+			+		Single	+		
45-03	29	+		+		+				+		Single	+		
4-08	46	+		+		+						Single			
49-07	48	+		+		+	+			+	+	Single			
121-09	49	+			+	+	+				+	Single			
19-04	49	+			+	+						Single	+		
50-09	58		+			+	+			+		Single	+		
35-05	60		+			+						Single			
166-05	68		+			+	+					Single	+		
71-10	17	+			+							Double			+
20-10	23	+			+							Single	+		
77-10	24	+		+								Single			+
94-08	24	+		+								Double		+	
115-10	27	+			+							Single			
101-09	27	+		+								Double		+	+
145-10	27	+		+								Double			+
123-95	28	+						+				Double			
3-11	28			+				+				Double			+
116-10	29	+			+							Single			+
122-09	38	+		+								Double		+	
49-10	39	+			+							Double		+	
64-08	40	+										Double		+	
140-10	40	+			+						+	Double		+	
25-07	43	+		+							+	Single			
123-09	44	+			+							Single			+
05-06	46	+		+							+	Double		+	
75-06	47	+									+	Double		+	
14-11	48	+		+				+				Double			
72-06	49	+		+							+	Double		+	
10-10	59		+								+	Single			
4-12	59		+								+	Single			
83-05	66		+									Single	+		

Table S2. Antibodies and their application

Target human protein	Antibody	Company	Application
CD49f (α6-Integrin)	(clone GoH3) Rat monoclonal	BD	Flow cytometry
EpCAM	(clone 9C4) Mouse monoclonal	BioLegend	Flow cytometry
CD31	(WM59) Mouse monoclonal	Biolegend	Flow cytometry
CD45	(clone HI30) Mouse monoclonal	Biolegend	Flow cytometry
hTERT	(clone Y182) Rabbit monoclonal	Millipore	Immunofluorescence (Confocal imaging)
TRF2	(clone 4A794) Mouse monoclonal Rabbit polyclonal	Millipore Santa Cruz	Immunofluorescence (Confocal imaging)
NBS1	(clone Y112) Rabbit monoclonal	Millipore	Immunofluorescence (Confocal imaging)
MRE11	Rabbit polyclonal	Millipore	Immunofluorescence (Confocal imaging)
RAD50	(clone 13B3/2C6) Mouse monoclonal	Millipore	Immunofluorescence (Confocal imaging)
γH2AX	Rabbit polyclonal	Millipore	Immunofluorescence (Confocal imaging)
53BP1	(clone BP13) Mouse monoclonal	Cell Signaling Technology	Immunofluorescence (Confocal imaging)
53BP1	Rabbit polyclonal	Millipore	Immunofluorescence (Confocal imaging)

Table S3. Primers used for Quantitative (q)Real-time PCR analysis

Target human mRNA	Primer pair used
hTERT	Forward, 5'-CGGTGTGCACCAACATCTAC-3'<br>Reverse, 5'-CACACATGCGTGAAACCTG-3'
MRE11	Forward, 5'-TGTTGGTTTGCTGCGTATTA-3'<br>Reverse, 5'-CATTCTTCTCTGCGGTTTGA-3'
RAD50	Forward, 5'-GTCATGTTGCCCCGTTTGTC-3'<br>Reverse, 5'-TTCTCTTGTTTCTCCTGGTT-3'
ATM	Forward, 5'-CAGGGTAGTTTAGTTGAG GTTGACAG-3'<br>Reverse, 5'-CTATACTGGTGGTCAGTGCCAAAGT-3'
ATR	Forward, 5'-ACATTCCCTGATCCTACATCATG-3'<br>Reverse, 5'-TTCAATAGATAACGGCAGTCCTG-3'
BLM	Forward, 5'-GGATCCTGGTTCCGTCCGC-3'<br>Reverse, 5'-CCTCAGTCAAATCTATTTGCTCG-3'
RAP1	Forward, 5'-GGGAGTTTGAGGAGGTTGTG-3'<br>Reverse, 5'-GGCTGTGTTTCTGAGTCTTC-3'
DNA-PK	Forward, 5'-CTTTGTCGTGTGGAGGGAT-3'<br>Reverse, 5'-CACAACGGGGTTCAGAAGTT-3'
TBP	Forward, 5'-TGAATCTTGGTTGTAAACTTGACC-3'<br>Reverse, 5'-CTCATGATTACCGCAGCAAA-3'
TBX2	Forward, 5'-CCCTTCCCGTTCCACCTC-3'<br>Reverse, 5'-ACTAGTGGCGGGCAAAGC-3'
TBX3	Forward, 5'-TCCATGAGGGTGTTTGATGA-3'<br>Reverse, 5'-CCATGCTCCTCTTTGCTCTC-3'
$P14^{ARF}$	Forward, 5'-CCCTCGTGCTGATGCTACTG-3'<br>Reverse, 5'-CATCATGACCTGGTCTTCTAGGAA-3'
P53	Forward, 5'-AGAGTCTATAGGCCCACCCC-3'<br>Reverse, 5'-GCTCGACGCTAGGATCTGAC-3'
ID2	Forward, 5'-GACCACCCTCAACACGGATA-3'<br>Reverse, 5'-CACACAGTGCTTTGCTGTCA-3'
E2F1	Forward, 5'-GGATTTCACACCTTTTCCTGGAT-3'<br>Reverse, 5'-CCTGGAAACTGACCATCAGTACCT-3'
PML	Forward, 5'-CAAGAAAGCCAGCCCAGAG-3'<br>Reverse, 5'-GTGCGCCAGGTGGTAGCTC-3'
